# 

**Published:** 1821-04

**Authors:** 


THE LONDON

### Medical and Physical Journal.

4 OF VOL. XLV.] APRIL, 1821. [NO. 266.

### NOTICE.

*The Proprietors acquaint the Medical Profession, that the* GENERAL INDEX *to this Journal is published, comprising an Analytical Table of its Contents, from* VOLUME ONE TO FORTY *inclusive; carefully arranged in Alphabetical Order, with References to the whole of the cited Authorities, under their nominal Characters, &c. and forming an indispensable* APPENDIX *to the Journal; in one large* 8*vo. volume, price* 21*s.*

### MONTHLY CATALOGUE OF MEDICAL BOOKS.

Home, on Strictures of the Urethra, &c. Vol. III. 8vo.

Transactions of the Association of Fellows and Licentiates of the King's and Queen's College of Physicians in Ireland. Vol. III. 8vo. 14s. boards.

Elements of Chemistry. By James Millar, M.D. 8vo. 12s. boards.

A Treatise on Cataract; intended to determine the Operations required by different forms of that Disease, on Physiological Principles. By Philip Chilwell de la Garde, Member of the Royal College of Surgeons, &c. 8vo. 8s. boards.

A Treatise on the Management of Female Complaints. By Alexander Hamilton, M.D. With Hints for the Treatment of the principal Diseases of Infants and Children; by Dr. James Hamilton, jun. 8vo. 10s. 6d. boards.

[HIGHLEY AND SON, FLEET-STREET.]

